# Graph SLAM Built over Point Clouds Matching for Robot Localization in Tunnels

**DOI:** 10.3390/s21165340

**Published:** 2021-08-07

**Authors:** Carlos Prados Sesmero, Sergio Villanueva Lorente, Mario Di Castro

**Affiliations:** Mechatronics, Robotics and Operations (SMM-EN-MRO), European Organization for Nuclear Research, 1217 Meyrin, Switzerland; sergio.villanueva@star-robotics.com (S.V.L.); mario.di.castro@cern.ch (M.D.C.)

**Keywords:** calibration, environmental reconstruction, graph SLAM, point cloud, registration, robot surveillance, robotic platform, self location

## Abstract

This paper presents a fully original algorithm of graph SLAM developed for multiple environments—in particular, for tunnel applications where the paucity of features and the difficult distinction between different positions in the environment is a problem to be solved. This algorithm is modular, generic, and expandable to all types of sensors based on point clouds generation. The algorithm may be used for environmental reconstruction to generate precise models of the surroundings. The structure of the algorithm includes three main modules. One module estimates the initial position of the sensor or the robot, while another improves the previous estimation using point clouds. The last module generates an over-constraint graph that includes the point clouds, the sensor or the robot trajectory, as well as the relation between positions in the trajectory and the loop closures.

## 1. Introduction

The objective of simultaneous localization and mapping (SLAM) is to provide both an estimation of the robot’s pose and a map of the unknown surroundings. This is particularly important during the inspection process in long tunnels. To this aim, we propose a fully original algorithm of graph SLAM that may be used in all the robots that have a set of minimum requirements and require a sensor that generates point clouds.

One of the most significant problems in underground tunnels is the survey of the proper performance of the security sensors available all along the whole corridor. The inspection of large tunnels can be laborious when performed by operators. However, robots may help with performing 4D tasks (Dirty, Dangerous, Difficult, and Dull), reducing risks for personnel. Inspection in underground tunnels was previously complicated because of (a) the long time to access the facilities, (b) the long time to escape the facilities in case of evacuation, (c) the strong safety protocols, and (d) the lack of GPS signal for localization. Although GPS is not available, the construction of the tunnels presents characteristics that can be found on the map, such as separating doors, entrances, exits, etc.—essentially, landmarks that allow the system to correct previous estimation errors.

A specific case is that of SPS (Super Proton Synchrotron), a particle accelerator that is currently the second largest machine of CERN’s (European Organization for Nuclear Research) accelerator complex. With a circumference of approximately seven kilometers, the corridor of the tunnel becomes completely monotonous, making navigation and SLAM an authentic challenge. Radiation sensors, which steadily measure the radiation, are located at variable distances in the ring. These sensors are essential to guarantee material and personal security. Due to the necessity of periodic check-ups of the sensors, inspections have to be carried out every month. During its journey, the robot described in [1] carries a radiation sensor to perform the radiation measurements.

Both radiation measurements, the one taken by the robot and the other taken by the fixed sensors, have to match. The result of this comparison has to be checked, in order to find faulty sensors. When both measurements taken in the same place are different, it is possible to determine where the damaged sensor is located. Thus, a robot needs to be sent to the specific point to fix the problem. For this reason, it is highly recommended to have an accurate system that estimates the position of the robot during surveillance.

The paper is organised in different sections. In Section 2, we discuss the different SLAM approaches. In Section 3, we describe the main components of the algorithm, their structure, and features. In Section 4, we explain the guidelines of the SLAM algorithm, as well as the logic and the functions of each part. In Section 5, we include two different options to calibrate a LIDAR, which allow finding the relationship between the reference system of the robot and the reference system of the sensor, even though the robot suffers modifications after its design. In Section 6, we finally discuss the results with two different sensors within two different environments.

## 2. Related Work

SLAM technologies equipped with various sensor systems, such as vision, laser, and ultrasonic sensors, have been proposed; however, they still face a number of practical issues, particularly in indoor environments [2]. Most systems use filter-based approach, such as EKF-SLAM, UKF-SLAM, and IKF-SLAM [3], or particle filters, such as MonoSLAM [4] or COP-SLAM [5]. When the computing resources are limited, it is possible to implement graph-based approaches, bundle adjustment-based approaches, or to combine them if sufficient computational resources are available.

In order to solve the problem of graph-based approaches, the authors in [6,7,8] presented non-linear optimization on constraint graphs. They successfully minimized the error produced by the inconsistencies between the position of the vertices and the constraints defined in the edges of the graph. Wagner et al. [9] combined these techniques with a principled way of handling non-Euclidean spaces and 3D orientations, based in particular on manifolds to build a framework. Olson et al. [10] presented a fast non-linear optimization algorithm that rapidly recovered the robot trajectory, even when given a poor initial estimate.

Grimes et al. [11] used a “hybrid Hessian” six-degrees-of-freedom SLAM, which incorporated GPS signals, and used stochastic SLAM methods, such as TORO [7] and Stochastic Gradient Descent SLAM, SGD. The Graph-SLAM-based algorithm requires a method of loop closure, such as [12], which presents an approach based on the iRRR algorithm. Ho et al. [13] proposed an algorithm that regularly captures with a camera and laser the appearance of the local scene. In addition, it detects the similarity between all possible pairings of scenes in a “similarity matrix” and, eventually, it poses the loop-closing problem as the task of extracting statistically significant sequences of similar scenes from this matrix.

Hess et al. [14] used a branch-and-bound approach for computing scan-to-sub-map matches as constraints in order to close the loop in 2D. Chen et al. [15] presented an algorithm that utilised a deep neural network exploiting different cues generated from LIDAR data for finding loop closures. This algorithm estimated an image overlap generalised to range images and provided a relative yaw angle estimate between pairs of scans. This approach provided good results in plenty of datasets; however, the feasibility of the system for implementation in low-feature environments, like tunnels, has not yet been demonstrated.

Another type of SLAM is vision-based SLAM, which might not operate well in environments with a low quantity of features and illumination changes, like tunnels. Visual SLAM may be feature-based (sparse, semi-dense, or dense) or intensity-based [16]. Most semi-dense SLAM techniques, like [17,18], rely on low-level characteristic features of the environment, such as corners, points, lines, and planes. These approaches typically deteriorate in performance in the presence of illumination changes and repetitive patterns. On the other hand, other state-of-the-art SLAM-based techniques, such as [19,20], focus on dense 3D mapping of the environment.

Lastly, other works combined IMU and RGBd odometry to localize the system within the environment, such as [21]. This presents a sparse visual odometry model for RGBd images. This technique utilizes the minimization of the photometric errors obtained from the edge features for pose adjustment to reduce the computational costs and to increase sturdiness.

In [22], Liu et al. presented a methodology to make visual odometry more robust through the use of a deep network model to predict complex camera motion. Using the image input and IMU output as an end-to-end training pair makes data collection cost-effective. In [23], Nieto et al. presented Scan-SLAM, whose approach is a marriage of EKF-SLAM and scan correlation. They defined landmarks using templates composed of raw sensed data. These templates can be augmented as more data become available so that the landmark definition improves with time.

## 3. Algorithm Components

**Figure 1:**
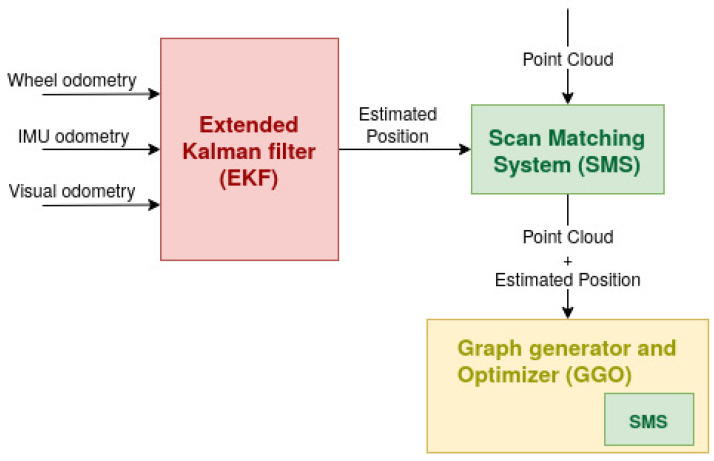
Structure of the SLAM system.

The responsible robot for the surveillance has to be localised within the environment to provide its position with the corresponding radiation measurement. The position in the ring is indicated in the main tunnel every ≈32 m with a very small posting sign. This fact, together with the high position where they are placed, makes it impossible to read the text of the signs and also comply with the time constraints. In order to achieve the project goals, another solution must, therefore, be proposed. The proposal SLAM system, shown in Figure 1, was implemented in the CERNTAURO framework [24], which contains all the software of the robotic team of CERN. It is composed of:

- An Extended Kalman Filter (EKF). This block combines odometric estimations in order to produce an estimated position. Odometry is to estimate the sequential changes of sensor positions over time [25]. EKF integrates three different odometry data sources: (a) the wheel odometry, which makes use of data from motion sensors (encoders); (b) the inertial odometry, which makes use of a inertial measurement unit, IMU, which is an electronic device that measures and reports a body’s specific force, angular rate, and the orientation of the body; and (c) the visual odometry, which consists of the process of determining the position and orientation of the robot by analysing the associated camera images. It is clear that the EKF works in open chain, accumulating errors in the pose estimation.
- A Scan Matching System (SMS). This block takes an estimated position as the input and improves it due to point clouds recorded at the same time as the position estimation. The methodology is implemented throughout the algorithm ICP (iterative closest point); however, other methods of 3D registration may be implemented. SMS, as well as EKF, works in open chain, and thus a chain closer should be implemented.
- A Graph Generator and Optimizer (GGO). This uses, as input, the SMS output (second estimated position). This block generates a graph SLAM problem, where each node is a position. When a position is well known (for example when a section door is identified), the loop closure comes into operation, and all the positions are corrected. Internally, this block makes use of the SMS.

As shown in Figure 1, the SLAM process begins with the Extended Kalman Filter (EKF), which is used for sensor fusion and non-linear state estimation. The output of EKF is the input of the SMS. This part compares two different point clouds, located in two consecutive positions. This module finds the relative pose (transformation) between the two point clouds taken. GGO has the objective of correcting the point cloud positions and, therefore, of improving the reconstruction and the location of the robot in the environment.

### 3.1. Extended Kalman Filter

EKF is the non-linear version of the Kalman filter, which linearises an estimation of the current mean and covariance. In the case of well-defined transition models, EKF has been considered the standard in the theory of non-linear state estimation, navigation systems, and GPS [26]. In this paper, we, therefore, propose a system that combines the information provided by the encoders, inertial measurements, and the visual odometry. The last one consists of a computer vision algorithm of feature detection, in order to detect and describe local features in images called descriptors.

By taking two consecutive images, it is possible to find the translation vector and the rotation matrix between both images, which may be used by the EKF as visual odometry. In each picture, the algorithm finds features, and then a matching system finds the correspondence between the features of both images. A random sample consensus (RANSAC) filter, is applied to the correspondences in order to remove the wrong ones that are out of range. Lastly, an error filter is applied, removing the correspondences whose average error is higher than a chosen threshold.

A comparison of feature detection algorithms can be found in [27], where the following algorithms are highlighted: (a) Direct Sparse Odometry [28] (DSO), (b) Speeded-Up Robust Features [29] (SURF), (c) Features from Accelerated Segment Test [30] (FAST), (d) Binary Robust Independent Elementary Features [31] (BRIEF), (e) HARRIS [32], (f) Oriented FAST and Rotated [33] (ORB), and (g) Scale Invariant Feature Transform [34], SIFT.

### 3.2. Scan Matching System

Scan matching, popularly known as registration, is an approach to recover the relative position and orientation of two laser scans. The iterative closest point algorithm (ICP) and its variants are the most well-known techniques for such problems. This set of non linear local searching algorithms presents several drawbacks, and therefore it is not common to use them without a previous alignment based on descriptors or another estimation source, such as robot odometry. Due to the non-convex optimization, if the estimated initialization is not sufficiently precise, the probability of falling in a local minimum increases and the convergence becomes slower [35].

As expressed in Algorithm 1, the typical stages of this kind of process include sampling or key-point selection, closest point searching for correspondence matching, wrong correspondence filtering, and point cloud registration making use of the optimal estimated transform. The main differences between ICP and descriptor-based registration algorithms are the sampling procedure and how the ICP iterates over the previous described stages until the convergence criteria is met.

**Figure 2:**
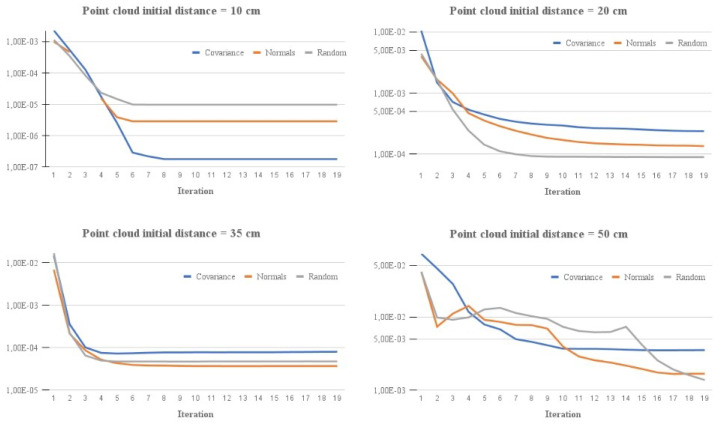
Sampling method comparison for different point cloud distances.

Key-point selection has a great impact on the speed and convergence of the algorithm. Among the sampling methods, it is possible to highlight uniform sampling [36], random sampling [37], normal space sampling [38], and covariance sampling [39]. Figure 2 shows a comparison between the different methods, with the peculiarity that the uniform and random methods provided very similar results and, thus, were condensed together.

The study of [40] compared and demonstrated that a combination between normal space sampling and covariance sampling methods provided the best convergence and stability results. Furthermore, when these methods were used, there was no need to execute them iteratively, thus, saving computational resources. However, we determined that, practically, there was no way to use those sampling methods due to the high execution times, which prevents using them online. Thus, we propose a combination between uniform and random sampling methods to achieve similar accuracy and faster execution times.
**Algorithm 1** ICP**Inputs:** Source(pA) and Target(pB) Point Cloud **Outputs:** Estimated transform $T^{*}$  **procedure**
ICP(pA,pB)    Na,Nb← NormalsEstimation(pA,pB)    $pA^{\prime },pB^{\prime }\leftarrow$ Sampling(pA,pB)    **while** covergenceCriteria=False **do**   c← CorrespondencesMatching($pA^{\prime },pB^{\prime },Na,Nb$)   $c^{\prime }\leftarrow$ CorrespondencesFiltering(*c*)   **if** $sizeof(c^{\prime })\geq 4$ **then**     T← TransformEstimation($c^{\prime }$)     $pA^{\prime }\leftarrow pA^{\prime }\times T$     ConvergenceEvaluation()    $T^{*}\leftarrow T$

Regarding the used transform estimation methods, research proved that the point-to-plane metric [41] highly improved the convergence and prevented the algorithm local minimum fall. Furthermore, there is also an advantage when using the point-to-plane metric in the presence of highly close surfaces, thereby, obtaining the best residual transform estimation results [42].

Other feature-based approaches, such as [43,44,45], rely on feature extraction in the point clouds. The key-point selection algorithm selected is ISS, and the descriptors are calculated making use of the FPFH algorithm, including the Persistent Features improvement. Summarizing, both systems are valid for our algorithm, which is able to switch between them easily due to their implementation with a common interface in the CERNTAURO framework [24].

### 3.3. Graph Generator and Optimizer

Thanks to EKF and SMS, it is possible to estimate the position of a taken point cloud with good accuracy. The GGO is an algorithm developed with the objective of achieving SLAM and, in this way, obtaining a good position of a robot in any kind of environment. Furthermore, this algorithm can be used to improve the reconstruction of the environment since the position of the different point cloud is optimised and improved. A general framework for (hyper) graph optimization (General Graph Optimization, g2o) was used to mount a graph and optimise it.

This is a C++ framework that performs the optimization of non-linear least squares problems that can be embedded as a graph or in a hyper-graph. This latter is an extension of a graph where an edge can connect multiple nodes [6]. Graph SLAM is an over-constrained problem that shall be solved by least-squares. Thus, the generated graph has the following basic features: (a) each node or vertex in the graph is a robot position (supposed by the SMS), (b) each vertex has an associated point cloud, (c) each edge in the graph corresponds with a relationship between two vertices, given by the internal SMS of the GGO, and (d) the graph is over-constrained, that is, usually each vertex has more than one edge connecting to other vertices.

The situation would be ideal if each point cloud of each vertex is compared with all the others, including the corresponding edge in the case that the correspondence between point clouds is found. However, this situation is impossible since the system must be implemented in a real-time application. A new position may be registered in the graph when the output values of the EKF are sufficiently large from the previous one in terms of the angle, translation, or time. This guarantees the uniform distribution of frames in the scene, thus, avoiding unnecessary repetitions and guaranteeing the correct correspondence between point clouds by the SMS.

It is known that a graph whose vertices are connected only with the previous and the following vertex is not optimizable since all the edge’s constraints are always satisfied. In order to solve this problem, the vertices shall be compared with the closest ones. Therefore, we distinguish two methodologies to find them: (a) the k-nearest neighbours methods, where each vertex is compared with the *k* closest vertices; and (b) the radius method, where each vertex is compared with the vertices that are within a circle of radius *R* that surrounds the position of the vertex (with a maximum of *k* neighbors to ensure the real-time feature).

In both cases, the system improves to the detriment of cumulative errors, since the results are more robust compared to errors when an over-constrained graph is created. Another advantage of this technique is the loop closure. When the robot is in a position where it was previously, the system generates a loop, which is used by the optimizer to correct the trajectory of the robot.

However, since the robot has to complete a turn in the accelerator complex with time constraints, it is expected that the robot does not come back to the previous positions, which would make it more difficult to close the loop in the graph. To solve this problem, another technique should be implemented. Since the accelerator door positions are well known, it is possible to detect them, to set their position as a fixed vertex in the graph, and to add the constraints between the robot and that position. Thus, since the origin position and the door position are well known in the large environment (fixed vertices), all the other vertices might be referred to the fixed vertices, and their position might be optimized.

The last point to be considered is the edge weights, namely to assign different importance to the constraints. They are reflected in the information matrix Ω, which is the inverse of the covariance matrix [6]. Its value may be determined by (a) scan matching the termination criteria, (b) the percentage of remaining points in the last ICP iteration if applied, or (c) the percentage of scan matching inlier points.

## 4. Algorithm Guidelines

**Figure 3:**
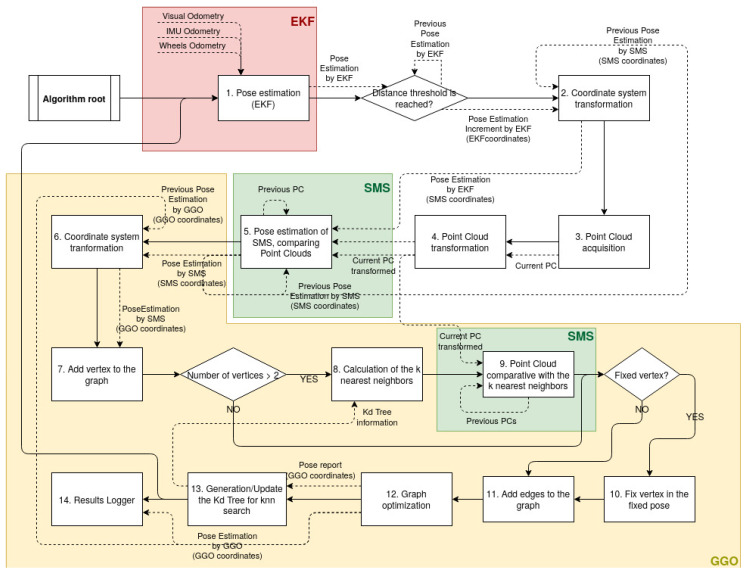
Flowchart of the graph optimization.

An algorithm was developed in order to solve the SLAM problem, whose flowchart is shown in Figure 3. In this paper, we define a coordinate as the position of the robot geometric centre from the point of view of an estimation system, such as the EKF, the SMS or the GGO. Thus, the position of the robot may be referred to one of the three different coordinates: (a) the coordinates of the EKF, (b) the coordinates of the SMS, or (c) the coordinates of the GGO.

With regard to the algorithm, point 1 “Pose estimation (EKF)” is responsible for estimating, in a loop, the robot’s first position. If the pose estimation in the previous iteration is far enough (the distance or the angle between two consecutive poses is larger than a threshold) from the current pose estimation, the system jumps to the next step.

**Point 2 “Coordinate system transformation”** consists of a frame change, which applies to the pose estimation increment estimated by EKF over the pose estimation in the SMS. With time, both systems differentiate since the accumulative errors change in both coordinate systems.

**Point 3 “Point Cloud acquisition”** consists of capturing a point cloud that is later used by the SMS. The driver of the sensor, which, depending of the application, may be the project budge or the design constraints is responsible for: (a) an RGBd camera, which is highly recommended in very small environments in applications where the design of the robot makes it more difficult to mount large components and when the budget is low; or (b) a 3D LIDAR, which provides a higher range, and, in this way it is easier to find the correspondence between point clouds in huge environments since more features will appear. The 3D LIDAR does not provide colour information, so the environment reconstruction would be colourless. Furthermore, the design has to allow a sufficiently large component, which is also expensive.

**Point 4 “Point Cloud transformation”** consists of transforming the point cloud from one frame to another one, namely a conversion between two coordinate systems (one located in the sensor that took the Point Cloud, and the other one located in the robot centre, where the pose estimation is calculated). This change can be made due to the transformation matrix being homogeneous, which is known due to the mechanical design or due to a calibration process explained in Section 5. Thus, the output of this block is a point cloud whose origin is located in the centre of the robot.

**Figure 4:**
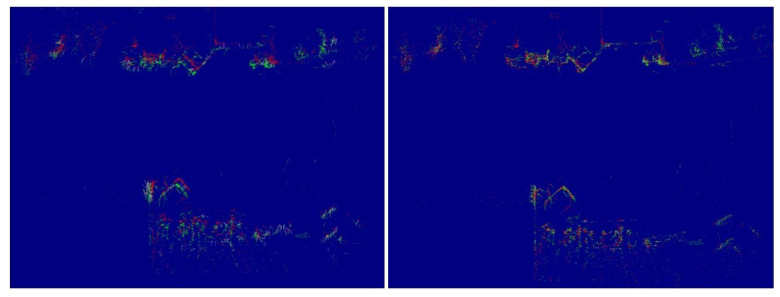
The first and last state of the point clouds when the SMS makes an estimation of the relative position between both. The red point cloud remains stationary, while the green one moves to reduce error.

**Point 5 “Pose estimation of SMS, comparing Point Clouds”** consists of comparing the current point cloud with the immediately previous one that is saved in the disk, by making use of the SMS. Figure 4 shows the initial state and the last state during the comparison between both point clouds (point clouds captured in a tunnel). It is possible to observe that the first estimation is not accurate, and, due to the SMS, both point clouds are aligned, thus, improving the accuracy. The output is a position and rotation estimation between the point clouds.

**Point 6 “Coordinate system transformation”** consists of a frame change, which applies to the pose estimation increase estimated by SMS over the pose estimation in the GGO.

**Point 7 “Add vertex to the graph”**. The new position and the corresponding point cloud are added to the graph in the form of a vertex. At this moment, the vertex is not connected to anything. If there are more than two vertices in the graph, the algorithm continues in point 8, otherwise, it jumps some steps.

**Point 8 “Calculation of the neighbors”**. If the number of vertices is higher than two, it is possible to find a correspondence between the current point cloud and some ones from other vertices (excluding the previous one that was already found). Previously, to come to this point, point 13 was already found, and thus a *k-d tree* is available in the system. Due to that, it is possible to explore the tree, looking for the nearest neighbours. At this time, it is possible to choose the mode (k-nn or radius search). The higher *k* that is selected, the more accurate and the slower the algorithm will be; therefore, the midpoint found (usually between 2–4), depends on the application.

**Point 9 “Point cloud comparative with the*****k*****nearest neighbours”**. At this point, the point cloud associated with the vertex (current one) is compared with the ones of the closest vertices. The result of this point is a set of pose transformation between vertices (constraints).

**Point 10 “Fix vertex to the fixed pose”** is enabled if the robot pose is known, e.g., at the beginning, when the robot is located in the charging station, or when it finds a landmark that is located in a known place (in this case, the main landmarks are the section doors).

**Point 11 “Add edges to the graph”** is responsible for adding the edges between the vertices. Since the last added vertex is disconnected, all its edges are added to the graph.

**Point 12 “Graph optimization”** is responsible for reducing the accumulative error and correcting the vertices poses when the loop is closed, or simply when the over-constraint graph may be corrected. This means that the sequence of robot pose is modified (vertices), keeping the edges with constants values. Since the library g2o is used for the optimization, a least-squares problem is solved. Thus, the quality of the system depends completely on the performance of the optimizer.

**Point 13 “Generate/Update the k-d tree for neighbours search”**. Once the graph is optimized, the pose of the vertices usually changes. The vertices pose in the graph is introduced in the k-d tree algorithm, which generates a tree with the position of the vertices (in *x*, *y*, and *z*), classified according to their values. It is later used by point 8, where the work of finding the nearest neighbours is significantly easier compared with other methods and extremely fast.

**Point 14 “Results Logger”**. In the last step, the results are saved in the disk. It is possible to save the point clouds, the environmental reconstruction point cloud (with the robot poses indicated in the form of red circles), a plot that shows the robot trajectory, and a file that details the raw data of the graph (vertices and edges).

In order to improve the quality results and to compute times, some fault tolerance techniques were developed. Thus, the following guidelines apply: (a) if the pose estimation increases by EKF and by SMS are too different (greater than a threshold), the SMS estimation is discarded; (b) the floor may be removed from the point cloud if desired (cutting it through a plane) in order to discard irrelevant information; (c) the points that are closer or farther than a threshold may be removed to avoid noisy points; (d) the estimations by the SMS when there is rotation out of the ’z’ plane may be discarded; (e) the estimations by the SMS when the fitness score of the matching process is lower than a threshold may be discarded; (f) the fitness score of the matching process is included as the weight of the graph’s edges; and (g) the output robot trajectory may be smoothed through a cubic spline. Point (a), (b), (d), and (e) are focused on avoiding the SMS falling in a local minimum.

## 5. Sensor Calibration

One of the most critical points along the implementation of the algorithm is the calibration of the sensor (used in point 4)—namely the estimation of the transformation between the robot centre, where the estimation of EKF is calculated, and the sensor, where the point cloud is captured. The design of the robot is sometimes accurate enough to skip this process. However, when some tests have to be performed in temporal, modular robots, or in robots that did not expect the sensor in the design, a calibration is needed. We, therefore, propose two different options to perform this calculation, each one with advantages and drawbacks.

### 5.1. Calibration through the Least Squares Method

The least squares method is a standard approach in regression analysis to approximate the solution of over-determined systems, i.e., where there are more equations than unknowns. The best solution is found by minimising the sum of the squares of the residuals made in the results of every single equation.

Applying this method, the robot is located in a known position within an environment with a high quantity of features (corners, walls, columns, etc.). The robot position is well known due to a camera located on the roof, which estimates its relative position according to a grid painted in the ground with a known size and structure. The robot moves to different positions where the 3D LIDAR takes a point cloud.

**Figure 5:**
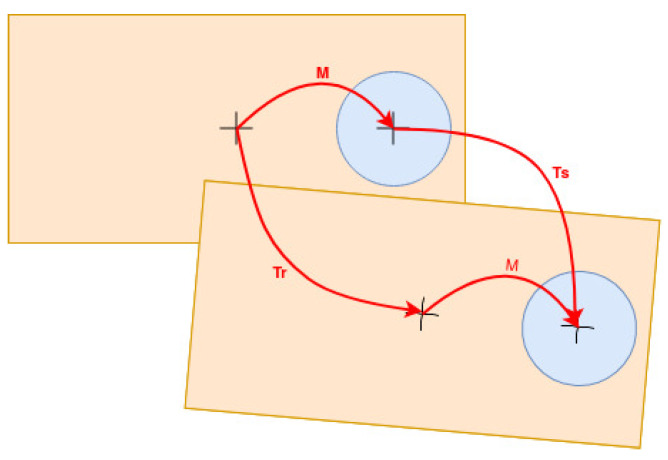
Transformation of coordinate systems in calibration using the least squares method.

Thus far, a vector of robot positions and point clouds are available. Offline, a developed software produces data in N−1 iterations, with *N* as the number of robot positions and point clouds. This data consists of (a) the transformation between two consecutive robot positions, $T_{r}$, and (b) the transformation between two consecutive point clouds due to the SMS, $T_{s}$. Both transformations help when the desired transformation between the robot and the LIDAR, *M*, is calculated, as shown in Figure 5, through the Equation (1).

(1) $$M\cdot T_{s}=T_{r}\cdot M$$

Thus, in each iteration, the components of the transformation *M* are calculated. However, since there are unavoidable mistakes in the estimation of the $T_{r}$ and $T_{s}$, the least square method is applied when these components are determined, reducing, as a maximum, the error of the estimation of the transformation *M*.

This method has several advantages: (a) it is not expensive as it does not need any specific equipment (it only requires a camera), (b) it is fast and accessible once the required software is developed, (c) the accuracy is good, (d) the spent time during the calibration is low when the operator is trained, and (e) it is generic for every point cloud sensor generator, both 3D and 2D.

However, this method has the following drawbacks: (a) it requires a lot of time to develop the needed tools, such as the software to estimate $T_{r}$ and $T_{s}$, (a computer vision program to identify the position of the robot, the SMS to estimate $T_{s}$, and the software to solve the least square problem), and the manufacture of the grid; (b) the first attempts of calibration require a considerable amount of time; and (c) the robot poses should be close to the previous ones to avoid wrong estimations by the SMS (if the ICP is used), since there is no initial estimation.

### 5.2. Calibration with Tracker Equipment

Tracker equipment is a machine that follows the trail or movements of a marker or markets, typically, to find them or note their course. In this case, a tracker, which estimates the position of a marker that is located in different spots and areas of the robot, can be used to find the relationship between the robot and the sensor.

In this paper, we propose a calibration process with the *Leica Absolute Tracker AT960* by *Leica Geosystems*, which is used in the *Survey group* of *CERN*. This product, together with the software *SpatialAnalyzer*, allows for the generation of planes, circles, points, etc., according to the path that the marker is following. Thus, the creation of planes in specific parts of the robot allows estimation of the robot centre where the EKF makes its calculations. In the same way, it is possible to move the marker through the sensor borders, as well as insert points to determine the orientation. The result is a 3D model with the locations of interesting data.

## 6. Results

**Figure 6:**
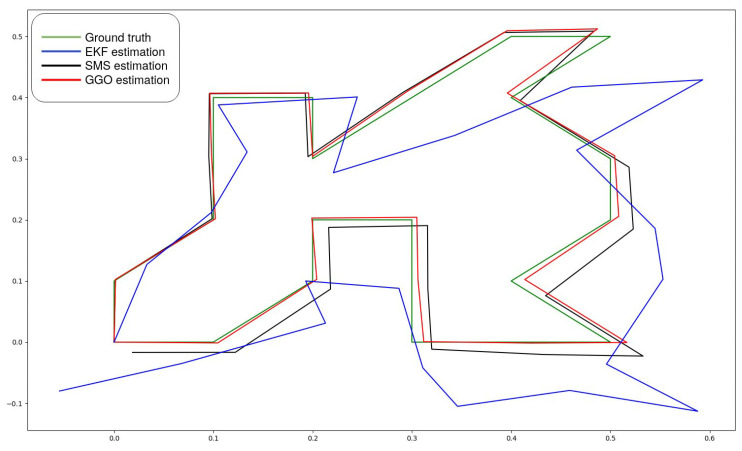
2D test results. The camera was moved in different steps on the ‘x’ and ‘z’ camera axes.

The first tests were carried out in small environments with the RGBd camera *D415* by *RealSense*. The results in one dimension (following a line) showed good performance even though the wheel odometry was inaccurate. The result in two dimensions (2D), shown in Figure 6, include the following details: (a) the pose estimation by EKF, denotes as a blue line, is separated from the ground truth line due to the cumulative errors; (b) the SMS output generated a good output; however, this signal accumulated errors that must be corrected; and (c) the GGO output followed faithfully the ground truth trajectory since the accumulative errors of the scan matching were reduced. In order to test the good performance of the system, a zigzag trajectory was followed by the robot, starting and finishing in the same point. The results showed good behaviour of the loop closure when the robot returned to previous poses and good behaviour when a known position was reached, thereby, correcting the followed trajectory.

**Figure 7:**
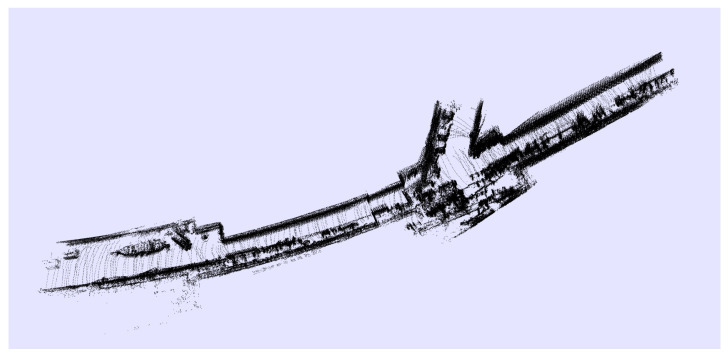
Reconstruction of the tunnel SPS during a survey. In this case, the area of test had many features to extract due to the lateral corridor and machines located in a wider area.

**Figure 8:**
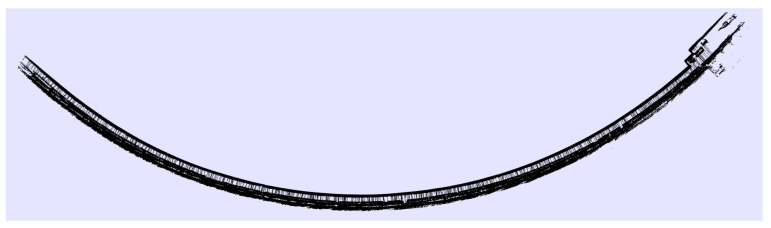
Reconstruction of the tunnel SPS during a long survey. In this case, the corridor was completely monotonous, excluding the right section.

**Figure 9:**
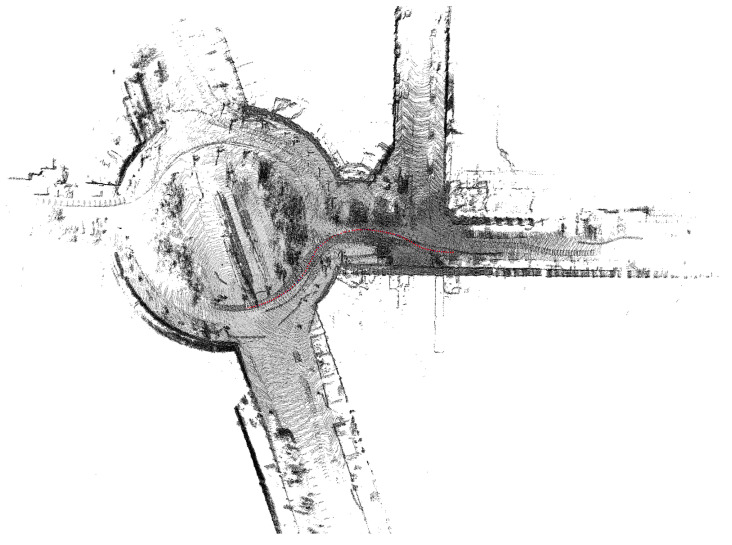
Test of the algorithm over the external point cloud dataset Kitti [46].

Going further, with an eye to the project requirement achievements, the system was tested in a huge environment, the SPS tunnel, which is a large place with low quantities of features for SMS, as shown in Figure 7 and Figure 8. The environmental reconstruction was produced correctly when the robot moved throughout the ring at a constant speed of 5.5 km/h (1.5 m/s). In these facilities with the corridor provision, other techniques based on 2D LIDARs or SONARs are completely useless, since they can not properly estimate where the robot is located with respect to the previous positions. The system was also checked within other environments, such as a city, obtaining results as shown in Figure 9.

**Figure 10:**
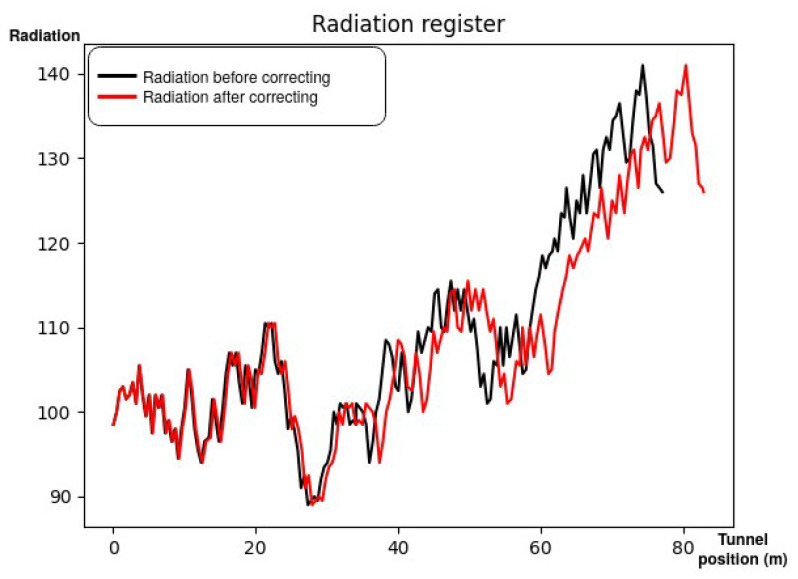
The radiation registered during a survey. The black line represents the radiation related to the position estimation by EKF, while the red line represents the radiation related to the position estimation by GGO, after finding a sector door at 82.81 m from the beginning.

Finally, in order to achieve the main goal (checking the radiation sensors along the ring), the results presented in Figure 10 show a comparison between the localization of a radiation measurement by EKF, where the errors are accumulative, and by GGO. Thus, it is possible to identify where the interesting radiation peaks are located.

This was measured that the position accuracy when the robot moved within a room of 25 m^2^ is ±0.25 m with the RGBd camera Realsense D415. If the used sensor was the detailed 3D LIDAR, the accuracy improved to ±0.12 m. Within a large environment, the RGBd camera showed poor performance, since its range was not enough to capture dense point clouds of the environment. If the used sensor was the LIDAR, the accuracy in a tunnel during a survey of 200 m (distance between accurate landmarks) was ±1 m.

## 7. Conclusions

In this paper, we presented original research done on Simultaneous Localization and Mapping (SLAM) for generic environments, and obtained good results in the location of robotic platforms in tunnels. We explained the main components of the algorithm, which used successive estimates of position to improve the final estimation. The system allows for the easy replacement of certain parts of the algorithm for others with similar capabilities, or even to remove them entirely, thereby, resulting in a modular system. The algorithm described can be used in robots that have a point cloud sensor, leaving the developer to choose the addition of the EKF (and its desired inputs) and the SMS technique.

In this way, we presented an algorithm with the ability to include new functionalities or to replace them, which provides great robustness, in addition to allowing easy integration into any type of system that has a sensor that generates point clouds from the environment. The proposed algorithm is able to localize a system in tunnel environments, where the lack of features makes the problem complex to solve. To achieve thsi, the only requirement that the system must have is a point cloud sensor generator.

Though the experiments, we demonstrated the good ability of environment reconstruction, even when the surroundings presented a low quantity of features, which made it more difficult, or even impossible, to use other SLAM approaches. In addition, we tested the behaviour of the algorithm during radiation monitoring and improved the localization of radiation peaks along the tunnel.

## Data Availability

Not applicable.

## Abbreviations

The following abbreviations are used in this manuscript:

SLAM	Simultaneous localization and mapping
CERN	European Organization for Nuclear Research
SPS	Super Proton Synchrotron
EKF	Extended Kalman Filter
SMS	Scan Matching System
GGO	Graph Generator and Optimizer
ICP	Iterative closest point
